# Exploring Patterns of Dynamic Size Changes of Lesions after Hepatic Microwave Ablation in an *In Vivo* Porcine Model

**DOI:** 10.1038/s41598-020-57859-1

**Published:** 2020-01-21

**Authors:** Keno K. Bressem, Janis L. Vahldiek, Christoph Erxleben, Franz Poch, Seyd Shnaiyen, Beatrice Geyer, Kai S. Lehmann, Bernd Hamm, Stefan M. Niehues

**Affiliations:** 1 Department of Radiology, Charité - University Medicine Berlin, Berlin, Germany; 2 Department of Surgery, Charité - University Medicine Berlin, Berlin, Germany

**Keywords:** Liver cancer, Cancer imaging, Targeted therapies

## Abstract

Microwave ablation (MWA) is a type of minimally invasive cancer therapy that uses heat to induce necrosis in solid tumours. Inter- and post-ablational size changes can influence the accuracy of control imaging, posing a risk of incomplete ablation. The present study aims to explore post-ablation 3D size dynamics *in vivo* using computed tomography (CT). Ten MWA datasets obtained in nine healthy pigs were used. Lesions were subdivided along the z-axis with an additional planar subdivision into eight subsections. The volume of the subsections was analysed over different time points, subsequently colour-coded and three-dimensionally visualized. A locally weighted polynomial regression model (LOESS) was applied to describe overall size changes, and Student’s t-tests were used to assess statistical significance of size changes. The 3D analysis showed heterogeneous volume changes with multiple small changes at the lesion margins over all time points. The changes were pronounced at the upper and lower lesion edges and characterized by initially eccentric, opposite swelling, followed by shrinkage. In the middle parts of the lesion, we observed less dimensional variations over the different time points. LOESS revealed a hyperbolic pattern for the volumetric changes with an initially significant volume increase of 11.6% (111.6% of the original volume) over the first 32 minutes, followed by a continuous decrease to 96% of the original volume (p < 0.05).

## Introduction

Microwave ablation (MWA) is a thermoablative procedure for the minimally invasive therapy of solid tumours, primarily in the lungs, kidneys and liver. It uses electromagnetic waves to induce local coagulation necrosis through heat^1–3^. During the ablation procedure, high local temperatures of up to 120 °C can be achieved in the target tissue, not only causing protein denaturation but also for example evaporation of the water contained in the tissue and resulting in changes of dielectric and thermal properties as well as structural tissue changes^4–6^. Overall, these structural changes can lead to dynamic alterations in lesion morphology and lesion size in the post-ablation period. Tissue shrinkage, primarily caused by dehydration, but also by tissue evaporation and carbonization, is an important change in the ablation area and ought to be considered in post-interventional imaging as it can cause a decrease in lesion volume of up to 30% and could therefore be of clinical relevance^7–9^.

Contrast-enhanced computed tomography (CECT) is often performed after MWA to determine the size and safety margin of the ablation area in order to ensure complete tumour ablation^10–13^. CECT has been shown to allow identification of the central necrotic area of the ablated tissue, which displays little to no contrast medium uptake and thus appears hypodense compared to normal liver tissue (NLT)^14^. Recent studies using CECT *ex vivo* or *in vivo* demonstrated the shrinkage of ablation zones in the first hours after MWA, but so far, no precise analysis of the dynamic size behaviour has been carried out^7,8,15^. However, precise knowledge of size could improve post-interventional assessment of ablation zones, as it also allows for evaluation of post-ablational shrinkage when evaluating sufficiency of the safety margin. Therefore, the present study aims to investigate the following aspects: firstly, to examine 3D volume changes of the ablation zones within the first hours after MWA; secondly, to analyse the patterns in size changes of different lesion areas within the ablation zone, and thirdly, to model the general lesion size behaviour over time.

## Results

MWA was performed in twelve anaesthetized pigs (154 ± 24 days, 79 kg ± 12 kg). Measurements at four different time points were performed for ten ablations in nine pigs; in three pigs, fewer than four measurements could be carried because the animals became unstable and the experiment had to be stopped. The median MWA duration was 15:00 min with a mean maximum temperature of 82 °C ± 44 °C. The average time between measurements was 13 (Interquartile Range (IQR): 20) min, more specifically, 20 ± 17 min between the first and second measurements, 14 (IQR: 5) min between the second and third measurements and 19 ± 9 min between the third and fourth measurement. Overall, 65 to 112 min passed between initial imaging immediately after the ablation and the final (fourth) imaging of the lesion

### Lesion morphology

**Figure 1:**
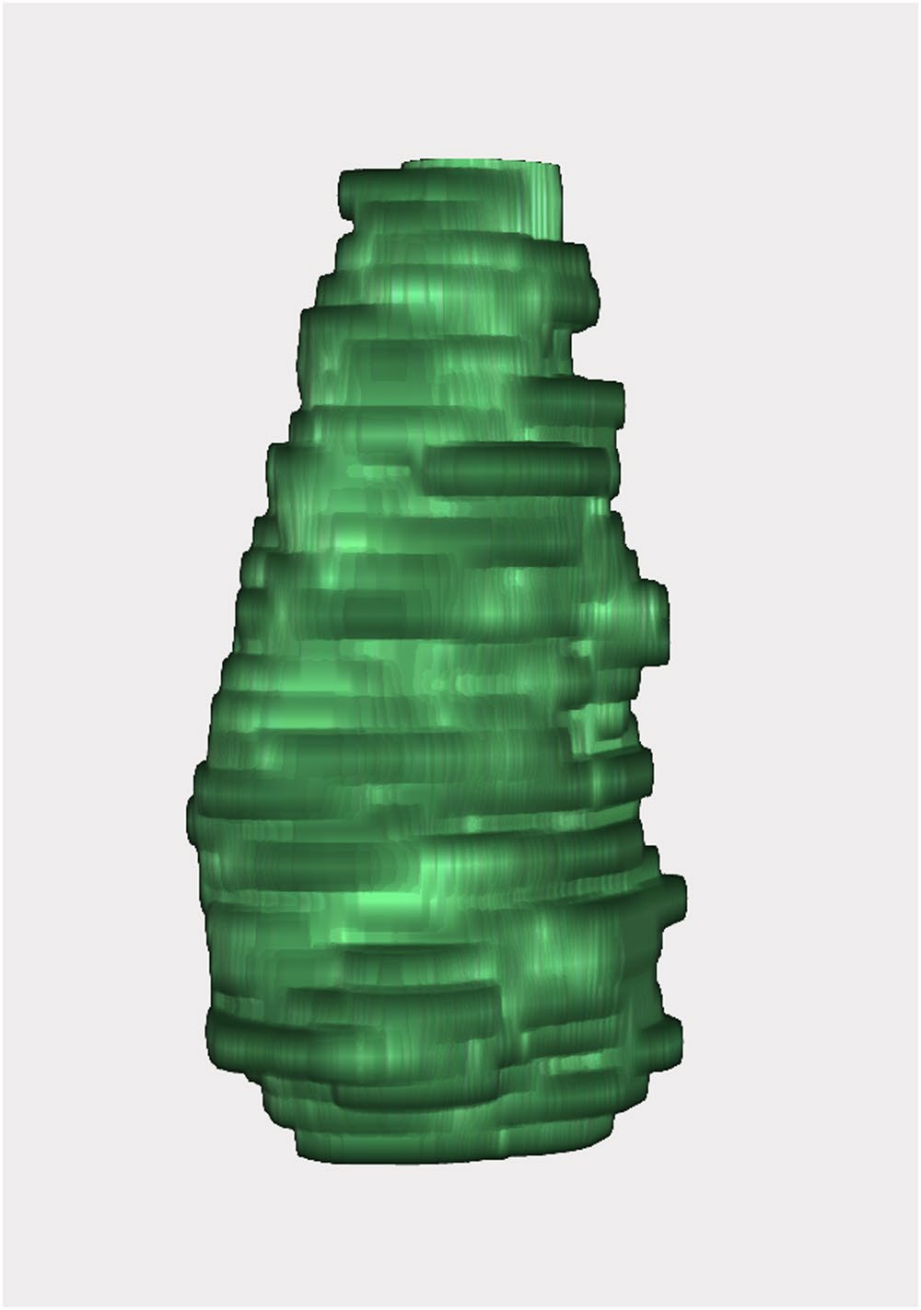
Example of asymmetric lesion configuration. Shows a lesion with the largest diameter at the lower pole and a smaller diameter at the upper pole instead of an overall spherical configuration. Similar shapes were found for most other lesions.

The lesions were predominantly ellipsoidal, with one lesion being predominantly spherical. They measured 52 ± 14 mm along the z-axis (first time point). Distally, around the tip of the antenna, the ablated lesions tended to have a larger diameter than at the proximal end (see Fig. 1 and Table 1). The inner region of the lesions appeared hypodense compared to the surrounding healthy liver tissue and was surrounded by a peripheral zone of increased contrast enhancement of highly variable intensity among lesions. A hypodense defect or a small hyperdense haemorrhage was observed centrally in the branch canal.

**Table 1:** Gives and overview of lesion locations, structural patterns and volumetric behaviour. It is noteworthy that lesions in close proximity to larger vessels showed more notches or bulges in their structures than lesions without adjacent vessels.

Lesion No.	Location	Structural pattern	Volumetric behaviour
1	Left hepatic lobe Close to the diaphragm Vessel at the lower pole	Small lesion predominantly oval	Steady decrease in volume
2	Left hepatic lobe Close to the diaphragm and stomach No adjacent vessel	Medium sized lesion predominantly oval	Hyperbolic behaviour, prolonged swelling at 4^th^ time point
3	Right hepatic lobe Close to diaphragm and gall bladder No adjacent vessel	Medium sized lesion Predominantly oval	Hyperbolic behaviour, overall decrease at 4^th^ time point
4	Centrally in the right hepatic lobe Small adjacent vessel at the lower pole	Medium sized lesion Oval with a central bulge	Hyperbolic behaviour, overall decrease at 4^th^ time point
5	Left hepatic lobe Close to diaphragm Central large vessels in frontal view and at the back of the lower pole	Small sized lesion Predominantly oval Central notch in frontal view (see Fig. 4)	Hyperbolic behaviour, overall decrease at 4^th^ time point
6	Centrally in the right hepatic lobe Two adjacent vessels dorsally of the lesion	Medium sized lesion elongated oval structure Two notches dorsally	Hyperbolic behaviour, Same volume at 1^st^ and 4^th^ time point
7	Centrally in the right hepatic lobe Adjacent vessel at the lower pole	Large lesion Predominantly round with bulge at the lower pole	Hyperbolic behaviour Prolonged swelling at 3^rd^ time point
8	Right hepatic lobe Close to the diaphragm No adjacent vessel	Large lesion Predominantly round	Hyperbolic behaviour Strong decrease in volume at 3^rd^ time point
9	Centrally in the right hepatic lobe No adjacent vessel	Medium sized lesion Predominantly oval (see Fig. 1).	Hyperbolic behaviour, overall decrease at 4^th^ time point
10	Left hepatic lobe Close to the diaphragm No adjacent vessel	Medium sized lesion Elongated oval structure (see Fig. 2)	Hyperbolic behaviour, moderate volumetric increase at 2^nd^, but strong decrease at 4^th^ time point

Also, lesions located close to the diaphragm tended to display more marked volumetric changes.

### 3D assessment of size dynamics

**Figure 2:**
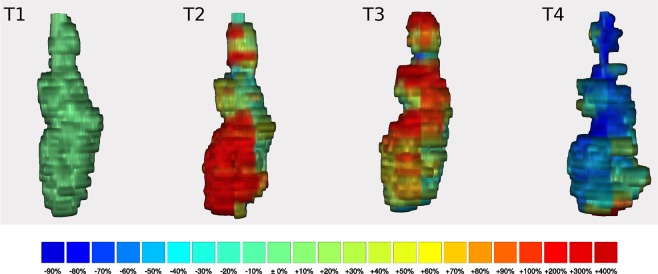
Example of a 3D reconstruction. Shows 3D reconstructions of lesion number ten in the frontal view over four consecutive time points (**T1–4**). The reconstruction of the first measurement is on the left (**T1**), followed from left to right by the second, third and fourth measurement (**T2–4**, respectively). The colour scale always refers to the percentage change in size compared to the previous measurement, with blue indicating shrinkage and yellow, orange and red indicating a slight, moderate and marked increase in size in the respective area. The colours indicate the local size changes and not the overall size change. Some small areas show variable size changes which did not seem to follow the overall pattern with selectively emphasized shrinkage of up to 80%, or a renewed increase of the volume, as for example indicated by the red colouration in the lower pole at time point four (**T4**). For the lesion shown here, these size dynamics led to a total volume decrease of 24% at 92 min compared with the original lesion volume.

The mean volume of lesions in the first measurement (immediately after ablation) was 15 ± 10 cm³, followed by an increase in volume in the second to third measurement and a subsequent decrease in the following measurements. 3D analysis revealed random size changes in the marginal lesion areas with small increases and decreases in volume of the lesion’s subsections, which, however, seemed not to contribute substantially to the overall initial increase and subsequent decrease in lesion volume. By contrast, we observed more pronounced dynamics of volumetric changes at the upper and lower poles of the lesion (defined as the upper and lower 25% of the lesions in the z-axis). Here, the lesion inflated and subsequently deflated over time following ablation. A noteworthy example of this process is given in Fig. 2. The parts of the lesion located centrally alongside the z-axis, however, seemed to have a more constant size dynamics with slower but constant shrinkage. The mean volumetric increase of the lesion poles after 32 min was 14% for the upper pole and 12% for the lower pole, while the central parts showed a smaller percentage increase of 5%. After 90 min, the upper pole volume decreased to 79% and the lower pole volume to 94% of the original size, while the central part showed a persistent minor swelling of 3%. Still, despite the asymmetric volumetric changes, the configuration of the lesion at the fourth measurement largely corresponded to that of the initial time point, so that the increase and decrease ultimately remained symmetrical.

### Modelling the size behaviour of lesions after MWA

**Figure 3:**
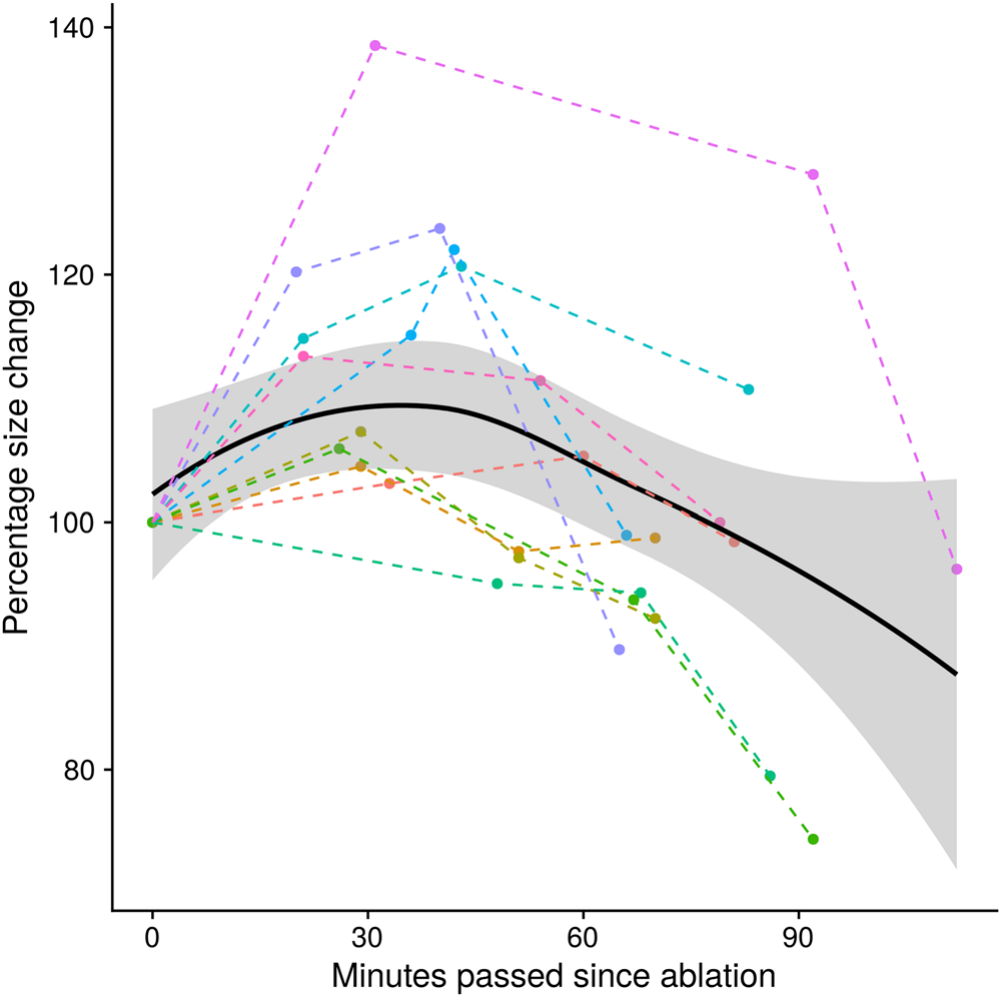
Overall size behaviour of all lesions. Shows the percentage size change starting from the first measurement (100%) over the time elapsed since the ablation procedure. The black curve was generated using a local polynomial regression model (LOESS) with the corresponding 95% confidence interval as grey area. This model shows an overall hyperbolic size behaviour of the lesions. One lesion shows more dramatic volumetric behaviour with an increase of nearly 40% at the first time point. This might in part be attributable to the overall small size of the lesion and its close proximity to a larger vessel.

After displaying the percentage change in volume compared to the elapsed time in a scatterplot, a locally weighted polynomial regression (LOESS) model (Fig. 3) was fitted on the data using a span of α = 1 for smoothing. The model showed an initial maximum increase in lesion volume to 111.6% (95% Confidence Interval (CI)116.6–106.5%) after 32 min with subsequent decrease. The lesions consecutively decreased in volume, with a small residual swelling after 60 min to 102.9% (95% CI 97.7–108%) of the initial lesion volume. The approximate initial volume was reached again after 63 min (95% CI 31–83 min), followed by a further decrease to 97.5% (95% CI 92.1–102.9%) after 75 min and to 96% (95% CI 89.3–102.7%) after 90 min. Altogether, the model shows a moderate fit to the data with a mean squared error (MSE) of 101.2 and a correlation of r = 0.58 (95% CI: 0.33–0.76, p < 0.001) between fitted values and original values.

## Discussion

The present study suggests that volume changes after MWA are more pronounced at the lower and upper poles of the lesion, although small random changes may occur throughout the entire lesion. Overall, lesions in our experiments showed an initial swelling up to 111.6% at 30 min after ablation with 32 minutes and a subsequent decrease to 96% of the original size after 90 min.

**Figure 4:**
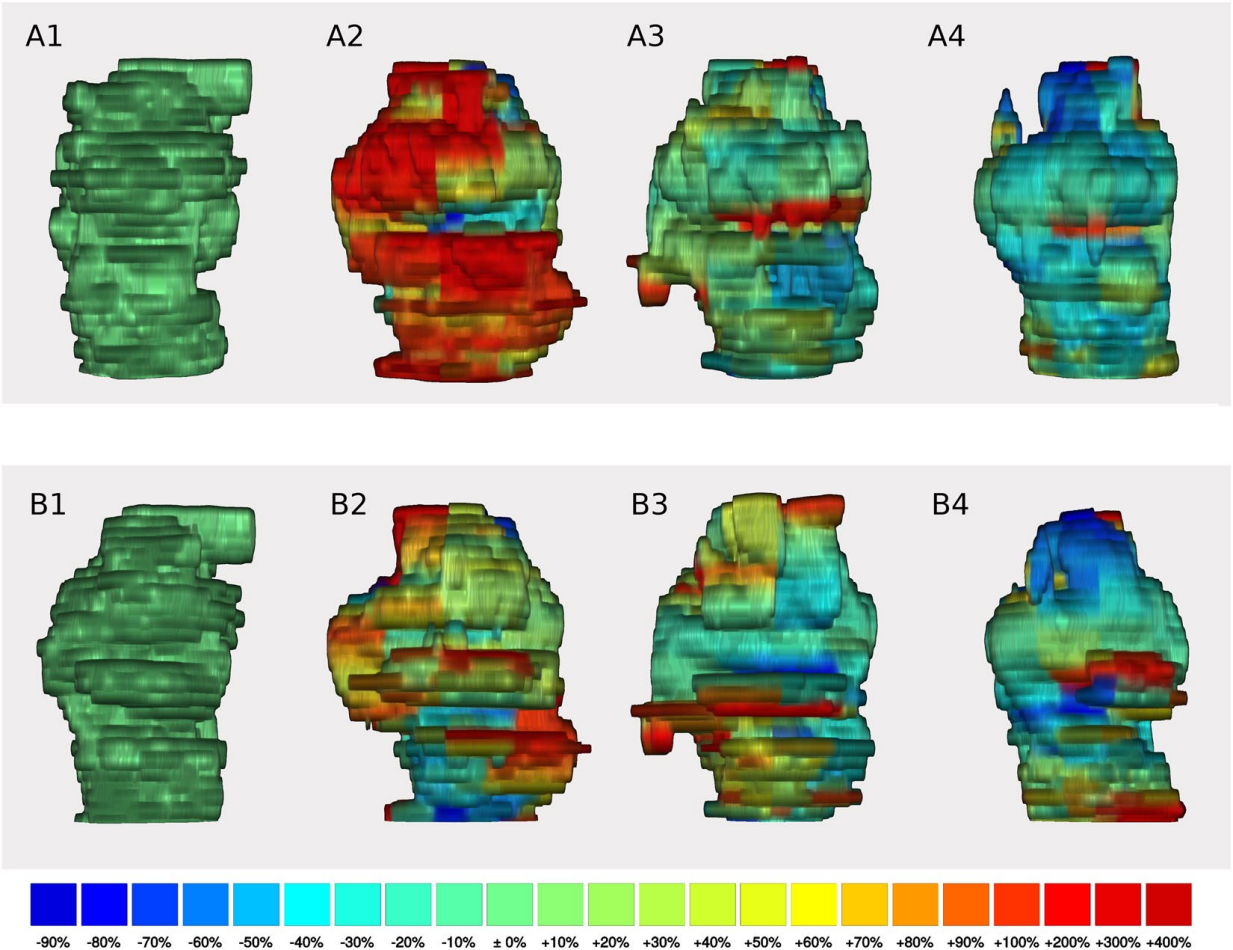
Example of a 3D reconstruction (lesion number five). Shows 3D reconstructions of lesion number five in the frontal view (**A1–4**) and in the rear view (**B1–4**). The reconstruction of the first measurement is on the left (**A1/B1**), followed from left to right by the second, third and fourth measurement (**A2/B2, A3/B3, A4/B4**, respectively). A strong but delayed increase in lesion volume can be detected in the third measurement (**A3**) adjacent to a vessel, which is indicated by the notch in the middle of the lesion in the frontal view. This increase in volume even continues in the fourth measurement (**A4**). Smaller size changes, with no clear pattern, can also be observed across time points. For example, a regional decrease in volume is seen at the back of the lesion in measurement 3 (**B3**), followed by a strong increase in the next measurement (**B4**) despite an overall decrease in lesion size.

The initial swelling of ablated lesions, as observed in the first half hour after MWA, is a surprising observation, which stands in contrast to the findings of some previous *ex vivo* studies^7,8,15,16^. An *ex vivo* study in bovine livers performed by Amabile *et al*. showed a linear correlation of shrinkage and time after ablation, while Brace *et al*. also demonstrated a linear decrease in the lesion size^7,8^. Conversely, in an *ex vivo* experiment, Farina *et al*. observed an increase in lesion size in the first minutes before subsequent shrinkage^17^. While they attributed the initial expansion to evaporation of tissue fluid during ablation and formation of gas bubbles^17^, it seems unlikely that this process would lead to a continuous lesion expansion for half an hour after ablation, as observed in the present study. Therefore, we assume that the protracted tissue swelling more likely results from osmotically active electrolytes, which are released from former intercellular spaces of the damaged cells during ablation and reactive oedema formation. However, volumetric dynamics differed between the individual lesions. Compared to other lesions, one lesion showed delayed dynamics with persistent swelling in the fourth measurement (lesion number two). In this case, our 3D analysis showed a largely homogeneous change over the entire lesion. The lesion was very close to the diaphragm, but no other possible influencing factors, such as large adjacent vessels, could be identified to explain the prolonged swelling. The most pronounced volumetric dynamics could be observed in lesion number five. On closer inspection in the 3D analysis, we identified an area through which a large vessel passed. This area showed a prominent initial swelling which only slowly receded and seemed to predominantly account for the lesion’s overalls volume changes (see Fig. 4 for 3D reconstructions).

**Figure 5:**
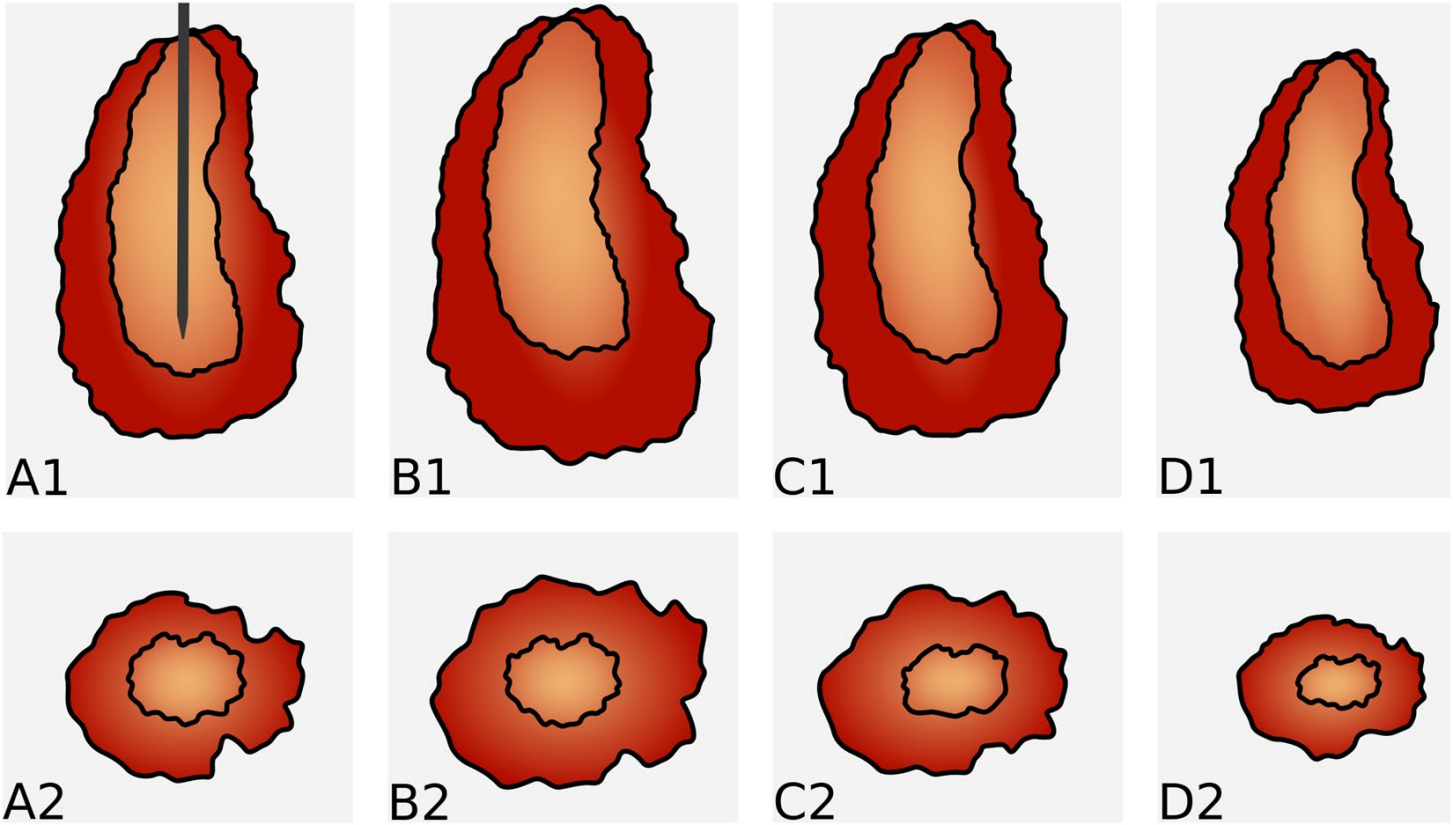
Schematic illustration of size changes. Shows a schematic representation of the size changes over four different measuring points, the top row along the electrode (**A1–D1**) and the bottom row in the cross section (**A2–B2**). Size changes mainly occur in the transitional zone, which provides a possible explanation for the asymmetric size behaviour.

It has been described that, macroscopically and histologically, MWA lesions can be divided into two zones, an inner zone, also referred to as white zone, and an outer transitional zone, also referred to as red zone^13,18^. While the inner white zone is completely necrotic, the transitional zone contains vital tissue with a partially preserved tissue structure. As the vascular structure may partially be preserved in the outer zone, this could be a plausible explanation for an increased influx of water from the surrounding tissue and the observed initial swelling. With increasing organization of the ablated area in the further course, the osmotically active molecules might then be degraded, resulting in lesion shrinkage, as conversion processes begin to dominate. Considering that all the above-referenced studies had *ex vivo* designs, this effect might not have been observed previously because dead tissues show less water and electrolyte exchange/reactive rebuilding compared to living tissues. However, further studies, especially with *in vivo* designs, are needed to support this theory. Studies with a 3D histopathologic reference standard, preferably with a nicotinamide adenine dinucleotide (NADH) vital stain, are considered particularly promising. The fact that the volume changes are more pronounced at the upper and lower poles may be due to the larger borders of the poles with healthy liver tissue compared to the central parts of the lesions, leading to a larger percentage of the transitional zone in this area (also see the schematic drawing in Fig. 5). The observed small, seemingly random changes in the peripheral area of the lesions probably result from a combination of inaccuracies in image segmentation, subtle motion artefacts and true small volumetric changes in the transitional zone.

A better understanding of the lesion size behaviour may help to improve MWA, since insufficient ablation might be more reliably identified with adequate consideration of size dynamics. In particular, knowledge of the initial swelling of the lesion within the first half hour, during which immediate control images are usually taken, would allow the interventionalist to directly perform additional ablation procedures whenever the initial ablation zone was too small. The initial swelling might thus mask incomplete ablation with the possible presence of residual tumour in the peripheral zone. Under the assumption that the volumetric changes primarily involve the transitional zone and considering the initial increase in volume, the chosen safety margin of ablations should be evaluated depending on the interval between the ablation and image acquisition. This implies that an observed safety margin of 5 mm, as currently recommended^19^, is adequate for image acquisition immediately after ablation, but might not be the optimal choice if imaging is performed later. In this case, as an increase in lesion volume might lead to an increase of the transitional zone to more than 5 mm, making the previously chosen safety margin insufficient. Rather, the extent of the safety margin should be chosen in dependence on the time passed since the ablation. For example, half an hour after ablation, the maximally measured increase in lesion size was 40%, corresponding to an individually adapted consequently the safety margin of should be adapted accordingly, which would correspond to 7 mm. In subsequent follow up (>24 h) imaging studies, lesion shrinkage, must be considered in evaluating whether complete ablation has been accomplished. However, our analysis suggests that the shrinkage is irregular, and, although the lesion poles appear to change more markedly in volume, it is not possible to deduce retrospectively, where the most dominant size changes have occurred. Therefore, a further reduction of the safety margin may be dangerous for the patient. Tissue shrinkage should also be considered for intermediate post-ablational imaging, when certain events cause a time delay. If the effect of shrinkage is not known to the performing interventionalist, this may lead to the false conclusion, that the ablation is inadequate. If this leads to re-ablation, this would cause unnecessary damage to liver-tissue and vessels. Another aspect is, that our analysis only covered a timeframe of up to two hours after the ablation. Therefore, the strong decrease in the second hour, as seen in Fig. 3, will likely weaken over time, making extrapolation of original lesion size from later post-ablation images challenging.

This study has some limitations. Due to the open cavity approach, hypothermia became more threatening with duration of the experiment. Therefore, the examination was usually stopped after the last ablation before the vital parameters became unstable. This might have resulted in a certain heterogeneity of the measurements and have reduced statistical significance due to the low numbers of measurements with four or more time points. We used domestic pigs and the properties of a healthy pig’s liver in terms of heat transmission could be different from those of a human liver containing cancerous tissue. Therefore, our results may not be fully transferable to humans with liver tumours. We used a low-power MW generator, which could have affected not only the lesion size but also the size dynamics and our findings are therefore probably not fully transferable to other devices. Compared to previous *ex vivo* studies, we also had longer ablation times, probably due to a lower power output from the MW generator as well as heat dissipation due to tissue perfusion and a heat sink effect near larger vessels. It is also possible that inflammatory processes have affected lesion dynamics, if one animal underwent multiple ablations. Taken together, these may have altered the volumetric changes of the lesions. As the LOESS model did not correct for possible clustering of the data, it might not be fully generalizable.

Overall, the lesion volume seems to follow a hyperbolic course after ablation with an initial swelling (over a period of half an hour) and a subsequent decrease to 96% of the original volume after four hours. Size dynamics are mainly attributable to volumetric changes at the upper and lower poles, although small random changes may occur throughout the entire lesion. One possible conclusion could be that the changes in volume are mainly attributable to osmotic processes of the partly vital tissue in the outer transitional lesion zone. Therefore, evaluation of the safety distance after ablation should be based on the time elapsed between ablation and imaging and, if necessary, the chosen margin ought to be adapted.

## Methods

### Animals, housing and care

MWA was carried out on domestic pigs in our facility. The animals were kept under controlled conditions in the stables of our facility and supplied with food and drinking water, in accordance with the local guidelines and rules for the implementation of the Animal Welfare Act. Prior to the interventions, the pigs were anaesthetized, and median laparotomy was performed. The study and all interventions were carried out in accordance with the guidelines and rules of the Federation of Laboratory Animal Science Associations (FELASA) and were approved by the National Office for Health and Social Affairs. This study was approved by the institutional review board of Charité - Universitätsmedizin Berlin.

### Microwave ablation

The electrodes for the microwave ablation (AveCure, MedWaves Incorporated, San Diego, USA) were inserted into the pig liver under CT fluoroscopy after the liver had been surgically exposed through a laparotomy. Ablations with post-ablational imaging were performed in an open cavity approach, resulting in a total of ten ablations with measurements at four consecutive time points, which were evaluated in this paper. Every MWA was performed for a median duration of 15 minutes with a power of 40 W. A target energy of 24 kJ was continuously delivered into the tissue during physiological blood flow and liver perfusion. After each ablation, a plastic tube was inserted into the centre of the lesion alongside the antenna to enable the reliable visualization of the lesion centre and the former position of the MWA antenna in the CT images.

### Contrast enhanced computed tomography

Delayed CECT scans were obtained after each MWA using an 80-slice multi-detector CT scanner (Aquilion PRIME, Canon Medical Systems Cooperation, Otawara, Japan) with the following parameters: 120 kV, 400 mA, mean mAs 260, collimated slice thickness 80 × 0.5 mm, 0.5 s rotation speed, pitch factor 0.814. The craniocaudal extension of the scanned area was 16 cm, covering the complete liver of the pig. For each scan, an iodinated contrast agent (100 ml) with a concentration of 400 mg I/ml (Imeron ® 400, Bracco Imaging, Milan, Italy) was administered at an injection rate of 4 ml/s.

### Data analysis

Images were exported as Digital Imaging and Communications in Medicine (DICOM) files in a 3D-image stack and imported into ITK-SNAP V 3.6.0 for semi-automatic segmentation of the lesion^20^. The first step was choosing a threshold density, which was held constant for every consecutive lesion. Next, the lesion was segmented using a region-growing algorithm. The hyperdense rim, which surrounded some ablations, was not included in the segmentation. Thereafter, 3D segmentations were imported into the “R” statistical environment (Version 3.4.0 - The R Foundation for Statistical Computing) as 3D arrays using the “oro.dicom” package^21,22^. Since the original images had slightly different size ratios, the arrays were first resized according to the pixel spacing stored in the DICOM header. For the analysis, the lesions were divided into 2D matrices along the z-axis and then further subdivided into eight subsections using the same segmentation mask for all lesions. Volumetric changes in these subdivisions were analysed qualitatively and quantitatively over the four time points and colour coded. The lesion volume was calculated from the pixel number and pixel spacing. Each colour-coded 2D matrix was exported into the uncompressed Tagged Image File Format (TIFF) and, subsequently, the generated image stacks were imported into ImageJ^23,24^. Surface meshes and 3D volume reconstructions were generated using the 3D visualization application programming interface (API) for ImageJ^25^.

### Statistical analysis

Statistical analysis was performed using the “R” statistical software environment Version 3.4.0 - The R Foundation for Statistical Computing) including the “ggplot2” package for graphical display^21,26^. Variables were expressed as mean ± standard deviation, if normally distributed, and as median (interquartile range - IQR) if not. To estimate the shrinkage, the percentage lesion volume was calculated in comparison to the first measurement taken directly after the MWA and correlated to the elapsed time. A locally weighted polynomial regression model was used to describe overall volumetric changes. Student’s t-tests were applied to assess the statistical significance of size changes over time. A p-value < 0.05 was considered statistically significant.

## Data Availability

The datasets generated and/or analysed during the current study are available from the corresponding author upon reasonable request.

## References

[1] Simon CJ, Dupuy DE, Mayo-Smith WW (2005). Microwave Ablation: Principles and Applications. RadioGraphics.

[2] Puijk RS (2017). Percutaneous Liver Tumour Ablation: Image Guidance, Endpoint Assessment, and Quality Control. Canadian Association of Radiologists Journal.

[3] Lucchina N (2016). Current role of microwave ablation in the treatment of small hepatocellular carcinomas. Annals of Gastroenterology: Quarterly Publication of the Hellenic Society of Gastroenterology.

[4] Lopresto V, Pinto R, Cavagnaro M (2014). Experimental characterisation of the thermal lesion induced by microwave ablation. International Journal of Hyperthermia.

[5] Vanni L, Rosanna P, Giorgio AL, Marta C (2012). Changes in the dielectric properties of *ex vivo* bovine liver during microwave thermal ablation at 2.45 GHz. *Physics in Medicine &*. Biology.

[6] Ai H (2012). Temperature distribution analysis of tissue water vaporization during microwave ablation: Experiments and simulations. International Journal of Hyperthermia.

[7] Brace CL, Diaz TA, Hinshaw JL, Lee FT (2010). Tissue Contraction Caused by Radiofrequency and Microwave Ablation: A Laboratory Study in Liver and Lung. Journal of Vascular and Interventional Radiology.

[8] Amabile C (2017). Tissue shrinkage in microwave ablation of liver: an *ex vivo* predictive model. International Journal of Hyperthermia.

[9] Sommer CM (2013). Quantification of Tissue Shrinkage and Dehydration Caused by Microwave Ablation: Experimental Study in Kidneys for the Estimation of Effective Coagulation Volume. Journal of Vascular and Interventional Radiology.

[10] Marin Daniel, Cappabianca Salvatore, Serra Nicola, Sica Assunta, Lassandro Francesco, D’Angelo Roberto, La Porta Michelearcangelo, Fiore Francesco, Somma Francesco (2015). CT Appearance of Hepatocellular Carcinoma after Locoregional Treatments: A Comprehensive Review. Gastroenterology Research and Practice.

[11] Kelekis A, Filippiadis D (2016). Computed Tomography and Ultrasounds for the Follow-up of Hepatocellular Carcinoma Ablation: What You Need to Know. Diagnostics.

[12] Bressem Keno K., Vahldiek Janis L., Erxleben Christoph, Poch Franz, Hiebl Bernhard, Lehmann Kai, Hamm Bernd, Niehues Stefan M. (2019). Instant Outcome Evaluation of Microwave Ablation With Subtraction CT in an In Vivo Porcine Model. Investigative Radiology.

[13] Gemeinhardt O (2016). Comparison of bipolar radiofrequency ablation zones in an *in vivo* porcine model: Correlation of histology and gross pathological findings. Clinical hemorheology and microcirculation.

[14] Vahldiek JL (2017). Characterization of benign periablational enhancement following multipolar radiofrequency ablation using perfusion CT in an *in-vivo* porcine liver model. Journal of Cellular Biotechnology.

[15] Farina L, Nissenbaum Y, Cavagnaro M, Goldberg SN (2018). Tissue shrinkage in microwave thermal ablation: comparison of three commercial devices. International Journal of Hyperthermia.

[16] Park CS, Liu C, Hall SK, Payne SJ (2018). A thermoelastic deformation model of tissue contraction during thermal ablation. International Journal of Hyperthermia.

[17] Farina L (2014). Characterisation of tissue shrinkage during microwave thermal ablation. International Journal of Hyperthermia.

[18] Geyer, B. *et al*. Microwave ablation zones are larger than they macroscopically appear - Reevaluation based on NADH vitality staining *ex vivo*. *Clinical hemorheology and microcirculation* (2019). 10.3233/CH-190583 31156148

[19] Kokudo N (2002). Genetic and histological assessment of surgical margins in resected liver metastases from colorectal carcinoma: minimum surgical margins for successful resection. Archives of surgery (Chicago, Ill.: 1960).

[20] Yushkevich PA (2006). User-guided 3D active contour segmentation of anatomical structures: significantly improved efficiency and reliability. NeuroImage.

[21] R Core Team. R: A language and environment for statistical computing. R Foundation for Statistical Computing, Vienna, Austria (2017).

[22] Whitcher, B., Schmid, V. J. & Thornton, A. Working with the DICOM and NIfTI Data Standards in R. *Journal of Statistical Software* (2011).

[23] Abràmoff MD, Magalhães PJ, Ram SJ (2004). Image processing with ImageJ. Biophotonics international.

[24] Schindelin J (2012). Fiji: an open-source platform for biological-image analysis. Nature methods.

[25] Schmid B, Schindelin J, Cardona A, Longair M, Heisenberg M (2010). A high-level 3D visualization API for Java and ImageJ. BMC bioinformatics.

[26] Wickham, H. *Ggplot2: Elegant Graphics For Data Analysis*, (Springer Publishing New York, 2016).

